# Building-Back-Better in Post-Disaster Recovery: Lessons Learnt from Cyclone Idai-Induced Floods in Zimbabwe

**DOI:** 10.1007/s13753-021-00373-3

**Published:** 2021-10-18

**Authors:** Ernest Dube, Gayan Wedawatta, Kanchana Ginige

**Affiliations:** 1 Department of Development Sciences, Faculty of Agribusiness and Entrepreneurship, Marondera University of Agricultural Sciences and Technology (MUAST), Marondera, Zimbabwe; 2 Department of Civil Engineering, School of Infrastructure and Sustainable Engineering, Aston University, Birmingham, B4 7ET UK; 3 Department of Architecture and Built Environment, Faculty of Engineering and Environment, Northumbria University, Newcastle upon Tyne, NE4 8ST UK

**Keywords:** Build-back-better, Cyclone Idai, Flood hazard, Post-disaster recovery, Zimbabwe

## Abstract

This study evaluated the build-back-better considerations in post-disaster recovery, following the devastation of Chipinge and Chimanimani communities by Cyclone Idai-induced floods in 2019. Conducted in 2020, the study assessed the impact of Cyclone Idai-induced floods on communities in Chipinge and Chimanimani Districts of Zimbabwe; evaluated the build-back-better considerations; and analyzed the lessons learned. Based on a qualitative approach and case study design, the study depended on focus group discussions, interviews, and researcher observations to gather data from 85 participants. The findings indicate that Cyclone Idai-induced floods seriously impacted human lives, infrastructure, and livelihoods of communities that had been living with flood risk and vulnerability. Build-back-better considerations were absent in much of the post-disaster recovery effort to address the cyclone disaster impact. There are important early lessons for both practitioners and community members to learn from the Cyclone Idai event. These lessons still can inform policy and disaster risk reduction practice in the medium and long term. Build-back-better should be a mandatory objective in the recovery from any disaster impact. Continuous training is also recommended to improve the disaster knowledge of stakeholders and increase local ability to cope with future disaster events.

## Introduction and Background

Building-back-better in the aftermath of major disasters, including cyclones, has often proved to be a major challenge to many governments and stakeholders. Evidence has shown that post-disaster recovery efforts taken without consideration of a build-back-better goal have often reconstituted the same pre-disaster conditions and vulnerabilities (Mannakara and Wilkinson 2014). For instance, Cyclones Idai and Kenneth have revealed the need to build-back-better due to unaddressed previous vulnerable conditions. Previous disaster recovery measures have tended to concentrate on just restoring communities to their pre-disaster state. Instead, post-disaster recovery, including reconstruction and rehabilitation, is an opportunity to not only restore communities (Khasalamwa 2009; Ozcevik et al. 2009), but also to create safer, sustainable, and more resilient communities underpinned by the concept of “build-back-better” (Clinton 2006). To build-back-better, governments, stakeholders, and disaster-impacted communities need to create long-lasting, resilient, and sustainable communities. Often recovery initiatives have failed to effectively restore disaster-impacted communities. For example, Wedawatta et al. (2018) noted that this was the case even after many years following the reinstatement of permanent housing. One of the reasons is that post-disaster recovery programs frequently must prioritize speedy restoration over disrupted system upgrades. Recovery should ensure the elimination of pre-existing vulnerabilities and increase resilience to future hazard events (Palliyaguru and Amaratunga 2008; Mercer 2010). True recovery creates resilient, safer, and more sustainable communities through building-back-better, because when future disasters occur, the built environment and social settings of communities are disrupted less severely (Dube 2020).

To achieve its aims, this study was guided by the following objectives: (1) assess the impact of Cyclone Idai-induced floods on communities in Zimbabwe’s Chipinge and Chimanimani Districts; (2) evaluate the build-back-better considerations in the Cyclone Idai post-disaster recovery; and (3) analyze the lessons learned from the Cyclone Idai-induced flood disaster.

In March 2019, Idai made landfall on the Mozambique coast and moved inland towards Zimbabwe’s Manicaland Province. In Mozambique the cyclone affected more than 1.5 million people, resulted in over 600 deaths, injured in excess of 1,600 people, and caused damage and loss estimated at USD 3.2 billion (Post Cyclone Idai Cabinet for Reconstruction 2019). In Zimbabwe, the cyclone triggered floods that killed many people, destroyed infrastructure, and disrupted livelihoods and social systems in the two districts of Chipinge and Chimanimani in Manicaland Province. More than 270,000 people and 17,608 households were left homeless with 341 casualties and many others missing (IFRC 2019). Rusitu Valley in Chimanimani is one of the flood-prone areas where Cyclone Idai left a trail of destruction (Chanza et al. 2020). The loss of human capital is a development concern, since people with skills and the ability to provide labor for sustainable development perished. The destruction of roads and bridges affected the mobility of the population, while damage to shelter and school infrastructure left many people homeless and halted access to education in the two districts. The recovery process that followed was not effective, because it lacked the build-back-better concept and focused on the quick restoration of disaster-impacted communities, which often may replicate or worsen existing vulnerabilities (Johnson et al. 2006; Lyons 2009). After Cyclone Idai, the recovery was characterized by weak disaster policies, poor structural designs, and inadequate reconstruction and rehabilitation measures.

If improvements are not taken, populations in Chipinge and Chimanimani Districts are likely to suffer the same impact from subsequent disaster events. Building the Chipinge and Chimanimani communities back better, beyond just recovery, is a necessity for effective disaster risk reduction. Simply rebuilding or restoring communities to their pre-disaster standards inherently recreates the same vulnerabilities that existed pre-disaster (Mannakara and Wilkinson 2014). Practitioners, stakeholders, and governments must reexamine their approach to disasters, apply the build-back-better concept, and save lives as well as improve infrastructure and livelihood.

## Review of Related Literature

This section examines the literature that was used to support the argument of the study. The literature consisted of media articles, published books, books chapters, journal articles, and online resources on disaster risk reduction and building-back-better. In this section, we present the theoretical framework that informs the study, which is guided by the Sendai Framework that highlights the essence of the building-back-better of communities in the post-disaster recovery phase.

### The Theoretical Framework: Sendai Framework for Disaster Risk Reduction

This study is informed by the Sendai Framework of the United Nations, which was adopted in March 2015 at the United Nations Third World Conference on Disaster Risk Reduction in Sendai, Japan. The Sendai Framework states that delegates “reiterated their commitment to address disaster risk reduction and the building of resilience to disasters with a renewed sense of urgency within the context of sustainable development” (UNISDR 2015, p. 9). The Sendai Framework calls for effective disaster risk reduction at national and local levels, with a strong institutional basis for implementation (UNISDR 2015). It has four priority areas, with Priority 4 focusing on enhancing disaster preparedness for effective response and building back better in disaster recovery, rehabilitation, and reconstruction (UNISDR 2015; UNISDR 2017)*.*

The authors believe that Priority 4 is important for disaster recovery processes in Chipinge and Chimanimani Districts, as it helps to foster the improvement of disaster-impacted communities. In line with the Sendai Framework, we stress that disaster practitioners can use modern scientific methods to attain build-back-better goals, while community members can use their indigenous knowledge systems to attain the same ends (Dube 2020). Indigenous people’s knowledge and practices complement scientific knowledge in disaster risk reduction (UNISDR 2015). In the Zimbabwean context, building-back-better means relying on the Civil Protection Unit and indigenous peoples’ capacities, strengths, skills, and resources.

The United Nations Office for Disaster Risk Reduction (UNDRR) (2017) defines build-back-better as “the use of disaster recovery, rehabilitation and reconstruction phases after a disaster to increase the resilience of nations and communities through integrating disaster risk reduction measures into the restoration of physical infrastructure and social systems and to revitalize livelihoods, economies and the environment.” In the African context, characterized by a lack of resources, build-back-better means restoration of disaster-affected communities through effective recovery, rehabilitation, and reconstruction for increased community resilience. Hence, the authors maintain that recovery processes in Chipinge and Chimanimani Districts based on the build-back-better concept can result in the proper restoration of infrastructure, livelihoods, and social systems in line with the Sendai Framework (Dube 2020). Meeting the challenge of the Sendai Framework goals is difficult because these objectives have not been fully embraced at the local level (Mavhura et al. 2020). Hence, post-disaster recovery measures may at times fail to materialize even though the Sendai Framework has been used as a guide.

### Impact of Cyclone Disasters and Post-Disaster Recovery Challenges

Cyclones are devastating natural hazards and they usually result in heavy flooding. The impact of cyclone-induced floods is at times so severe that the built environment, important assets, and community livelihoods are heavily damaged (Lin et al. 2015; Yan et al. 2016; Sadik et al. 2018). The huge impact presents many complications when it comes to implementing post-disaster recovery measures. The damage, losses, and disruption that cyclones cause have often caused communities and practitioners to exclusively promote rapid recovery. The failure of development initiatives to effectively achieve full recovery can recreate the same vulnerability conditions that caused the disaster (Mannakkara et al. 2018; Dube 2020). In such circumstances, post-disaster recovery programs are expected to address previous structural, community set-up, and legislative and policy imperfections, thereby creating enhanced improvement. Recovery through the build-back-better concept should offer windows of opportunity to enhance the resilience, sustainability, and reduced vulnerability of disaster-impacted populations (World Bank 2009; Hallegatte et al. 2018).

Cyclone Eline, which hit Zimbabwe in 2000, exemplifies a cyclone’s distructive power. Over 250,000 people were impacted, approximately 120 fatalities occurred, and 59,187 houses were damaged, in addition to economic losses estimated at USD 7.5 million (Shumba 2005). By impacting human capital, cyclones can retard progress towards achieving development, since able-bodied and skilled people can be numbered among the killed or injured.

In 2005, Hurricane Katrina severely impacted the Gulf Coast states of Louisiana, Mississippi, Florida, and Alabama in the United States, which led to the adoption of serious build-back-better measures, particularly in New Orleans. Hutton (2008) notes that during Hurricane Katrina approximately 1330 people were killed. The economic damage was estimated to exceed USD 170 billion (USGAO 2020). By causing such significant damage, Hurricane Katrina posed a threat to the development of the United States and indirectly impacted other nations that depended on the country for development aid. The 2007 Cyclone Sidr hit Bangladesh, and affected approximately 9 million people across 30 districts, resulting in around 4,000 deaths (Walton-Ellery 2009). In 2009 Bangladesh was again impacted by Cyclone Aila, which affected 152,496 people in Koyra (Sadik et al. 2018). Further, the cyclone caused damage to an 81 km stretch of flood embankments, 49 bridge culverts, 42,440 houses, nine academic and 192 religious institutions, 11,500 ha of crops, and 10,364 fish aquaculture farms (Sadik et al. 2018). The 2017 Hurricane Irma also heavily impacted infrastructure in Saint Martin by damaging electric, water, and telecommunication systems in addition to the disruption of transport networks (Nicolas et al. 2018). The severe impacts of the cyclones and repeated losses of life and infrastructure are enough evidence that effective restoration of communities needs programs with serious build-back-better considerations. Because the impact of cyclones poses a serious recovery, rehabilitation, and reconstruction challenge on the development of nations, post-disaster recovery needs a build-back-better emphasis to achieve improvement.

### Background of the Build-Back-Better Concept

The origins of the build-back-better concept have been misunderstood. Some scholars and practitioners regard it as a new concept, while others maintain that the practice has been there for some time. There has been some confusion about the phrase “build-back-better,” with the definition of the word “better” being interpreted in various ways (Kennedy et al. 2008). Some scholars have interpreted “better” to mean modernization, while others strongly suggest that the term “building-back-safer” is more appropriate because it focuses on structural safety in rebuilds (Kennedy et al. 2008). Building-back-better has been regarded as building-back-stronger, since it reduces losses associated with future disasters by ensuring that the reconstructed infrastructure can resist more intense disaster events (Hallegatte et al. 2018). Building-back-safer and stronger seem appropriate explanations of building-back-better, since they suggest improvement of the high risk and vulnerability conditions often existing in a pre-disaster state. What is clear about the build-back-better idea is that the concept has had much influence on current disaster risk reduction thinking (Lyons 2009). The need for effective recovery with a focus on building-back-better was given a United Nations mandate when the Sendai Framework was signed in 2015 (UNISDR 2015). But building-back-better gained popularity earlier during the large-scale reconstruction effort following the Indian Ocean Tsunami in 2004 (Mannakkara et al. 2018). The concept emerged specifically as a response to the need to improve recovery practices to build safer communities (Clinton 2006; Lyons 2009). We contend that enforcing build-back-better measures in post-disaster recovery processes after the devastating impact of Cyclone Idai in Zimbabwe can result in the building of safer and resilient communities.

Build-back-better has been understood as a holistic concept for using post-disaster reconstruction as an opportunity to improve the physical, social, and economic conditions of vulnerable communities (Khasalamwa 2009; Mannakkara and Wilkinson 2014). In terms of flooding, it also means the restoration of institutions and infrastructure that are better than those that existed before the recent disaster in Chipinge and Chimanimani Districts. Build-back-better also means the promotion of nonstructural measures, such as providing disaster risk reduction education (Mannakkara et al. 2018). The subsection that follows focuses on the benefits of building-back-better in post-disaster recovery.

### The Benefits of Building-Back-Better in Post-Disaster Recovery

The idea behind the build-back-better concept is to create stronger and more resilient communities following a disaster event (Mannakkara et al. 2014; Dube 2020); many communities have often faced the same fragile pre-disaster conditions. Kennedy et al. (2008) argue that rebuilding the built environment and infrastructure exactly as they were before a disaster often recreates the same vulnerabilities that existed earlier. Post-disaster recovery processes such as reconstruction and rehabilitation present an opportunity to address and rectify vulnerability issues found in disaster-impacted communities (Kijewski-Correa and Taflanidis 2012).

In the context of this study, vulnerability is defined as “the conditions determined by physical, social, economic and environmental factors or processes which increase the susceptibility of an individual or a community, assets or systems to the impacts of hazards” (UNDRR 2017). We know that such conditions exist in Chipinge and Chimanimani Districts. Communities in these districts need to assimilate that knowledge and develop a deeper understanding of their state of vulnerability, risk, and hazard in order to avoid future disasters. The build-back-better concept can create safer and more resilient communities and encourage construction of new buildings and facilities—infrastructure that never existed before. Manyena (2009) regards the post-disaster recovery period as providing “new things” such as new schools, new shelters, and improved health facilities. Dube (2020) views these as “recovery surpluses,” since they are add-ons that never existed before. In a nutshell, building-back-better presents an opportunity to fully recover from the present disaster impact, at the same time addressing risks and vulnerabilities associated with future hazards.

## Materials and Methods

This section outlines the materials and methods that were adopted in the study. It covers description of the study area, the research approach, sampling strategy, data collection techniques, and ethical considerations for the study.

### Description of the Study Area

The study was conducted between July 2020 and March 2021 in the districts of Chipinge and Chimanimani in Manicaland Province of Zimbabwe. The two districts share a boundary and are located in the eastern part of Manicaland Province. Chipinge District has a population of 324,133, grouped in 31 rural administrative wards and eight urban administrative wards, which are located in Chipinge Town (ZimStat 2013). Chimanimani District’s population stands at 134,940 and this population is 95% rural (ZimStat 2013). The districts are separated by the Save River and are flood-prone areas. Communities in these districts rely mostly on subsistence farming for livelihood. There are also commercial farmers in the districts who grow crops to feed the province and nation. Communities also maintain sugarcane and banana plantations along streams and wetlands, which are a source of household food security and nutrition. Research has revealed that the two districts, apart from being prone to flooding, are also subjected to severe drought (Bongo et al. 2018).

### Research Approach and Sampling

The study adopted a qualitative approach since the aim was to explore and learn from the experiences of Cyclone Idai-impacted community members and the practitioners who undertook post-disaster recovery programs. The qualitative research approach produces results mainly in the form of descriptive textual information (Kirton 2011). This research approach investigates issues such as people’s opinions, feelings, and values; interpretations and responses; behavioral patterns; process and patterns; and often employs case studies, including critical incidents (Kirton 2011). A case study design that incorporated the two districts informed the study, which was based on a purposive sample of 85 research participants—60 community members and 25 disaster risk reduction (DRR) practitioners. This sampling method helped to focus more on the flood survivors and practitioners with appropriate knowledge and experience of the phenomenon being studied.

### Data Collection Techniques

Both primary and secondary data were gathered to explore the problem of building-back-better in post-disaster recovery. The secondary data analyzed involved journal articles, special reports, books, and book chapters. This literature focused on disaster risk reduction, building-back-better, and post-disaster recovery. The study also considered publications on the Sendai Framework. This helped the researchers to construct new concepts and advance their theoretical framework (Noor 2008). To complement available secondary data, the study gathered primary data from the field using in-depth interviews and focus group discussions (FGDs) reported in Table 1.

**Table 1:** In-depth interviews and focus group discussions (FGDs) adopted for data collection

Research technique	Number of participants	Percentage (%)	Category of participants
Interviews	60	71	Community members
FGDs	24	29	Disaster risk reduction practitioners
Total	84	100	

In-depth interviews were administered to the 60 community members, while FGDs were used to collect responses from the 24 DRR practitioners. The observation technique was used to gather onsite, first-hand information about the destruction caused by the Cyclone Idai-induced floods, as well as information about the build-back-better programs in post-disaster recovery. Community members were chosen for their lived experiences, whereas the practitioners were chosen based on their experiences of managing Cyclone Idai-induced flood disasters. Community members were chosen indirectly through village heads, and the practitioners were chosen through District Development Coordinators and local humanitarian organizations. The practitioners included participants from Zimbabwe Republic Police (3), Zimbabwe Defense Forces (3), Ministry of Health and Child Care (3), Ministry of Local Government and National Housing (3), Agricultural Technical Extension Services (Agritex) (3), local nongovernmental organizations (NGOs) (6), and Ministry of Primary and Secondary Education (3). Data were analyzed based on the qualitative content thematic categorization, which transformed the data into meaningful findings (Patton 2002).

### Ethical Considerations

This research was conducted during the COVID-19 pandemic era. The government of Zimbabwe imposed movement restrictions and lockdown through Statutory Instrument 83 of 2020 (Zimbabwe Government 2020). When data collection was performed, however, the government had eased the movement restrictions and the researchers managed to conduct fieldwork. As such, ethical issues, including considerations for the COVID-19, were taken on board. Social distancing, the wearing of face masks, and hand sanitization were some of the ethical issues observed by the researchers and the research participants. The objectives of the research (Guillemin 2010) were explained to the participants, and informed consent (William 2006) was obtained. The participants were also assured of anonymity of identities and confidentiality of responses (Guillemin 2010). The participants also were informed that their involvement in the study was voluntary and that no rewards were to be offered.

## Results and Discussion

This part presents the results of the study as learned from the respondents in Chipinge and Chimanimani Districts. The results are further discussed in line with the research objectives and the themes developed from the analysis of data. Results from previous studies and the theoretical framework for the study—the Sendai Framework—were also used in the discussion. The following thematic categorization was used to present and discuss the results: Cyclone Idai-induced flood impact on communities in Chipinge and Chimanimani; evaluating the build-back-better considerations in Cyclone Idai post-disaster recovery; and, build-back-better lessons learnt from the Cyclone Idai disaster.

### Cyclone Idai-Induced Flood Impact on Communities in Chipinge and Chimanimani

The 2019 Cyclone Idai-induced floods in Chipinge and Chimanimani Districts resulted in huge devastation to communities. In-depth interviews with community members and FGDs with DRR practitioners revealed that the communities suffered massive losses in terms of the number of people killed, destruction to infrastructure, damage to property including shelters, environmental degradation, and community livelihoods, including livestock. These results are similar to a study about the 2004 Indonesia tsunami, which resulted in approximately 500,000 people losing their homes, and an estimated 750,000 people losing their livelihoods (Fan 2013). The losses incurred by the communities in Chipinge and Chimanimani Districts have severe implications, including for the districts’ endeavors to spearhead development. Table 2 presents a summary and analysis of the disaster losses obtained from the interviewees and FGD participants.

**Table 2:** Disaster losses impacting communities in Chipinge and Chimanimani

Disaster losses	Description of the losses	Loss implications or effect
Human life losses	A large number of people killed, some injured, others incurred disabilities	(1) Human capital with valuable knowledge and skills killed (2) People with disabilities cannot effectively contribute to community development (3) Household poverty is likely to increase
Infrastructure losses	Roads and bridges destroyed; school buildings and health centers damaged; grocery shops damaged; church buildings damaged	(1) Road network affected (2) No easy access to other districts and provinces (3) Children’s education disturbed (4) Good health and well-being compromised (5) Food security affected as grocery shops were impacted (6) Community spiritual needs impacted
Household property losses	Destruction to shelter; damage to household property such as furniture; loss of personal documents such as identifying documents, school leaving certificates (school diplomas), birth certificates, baby cards and birth records; loss of children’s school books and stationery; damage to cars, Scotch-carts (sturdy, two-wheeled carts drawn by an ox); damage to farming equipment	(1) People left homeless (2) No ownership of assets (3) Lack of personal identification (4) Children’s education disturbed (5) Vehicle ownership and travelling disrupted (6) Farming disturbed
Environmental losses	Destruction of timber plantations and forests; land degradation; destruction of wildlife species	(1) Timber loss is economic loss (2) Wildlife loss is economic loss
Community livelihood losses	Damage to water sources such as boreholes, dams, wells, and springs; damage to sugarcane and banana plantations; loss of livestock such as cattle, goats, sheep, and chickens; damage to estates, farms, and crop fields	(1) Water supply interrupted (2) Loss of livestock is economic loss (3) Damage to estates, farms, and crop fields are economic loss

*Source* Authors’ construction from field data, 2020−2021

Based on the information presented in Table 2, losses suffered from Cyclone Idai have many implications. Human life losses and injuries harm communities because people with knowledge and skills and labor productivity are lost or damaged. Such knowledge and skills are important and necessary for community attainment of development goals. Hence, building-back-better would ensure that loss of life due to cyclones and related disasters is reduced or prevented in the future.

Both the interview and FGD respondents indicated that the destruction of the built environment by cyclone-induced floods meant that their lives would never be the same again. Cyclone Idai impacted infrastructure that included roads, bridges, school buildings, churches, and shops. People’s movement was restricted, since they could not access other areas across rivers due to damaged bridges; children’s education was disrupted and local businesses could not supply basic commodities. Our results agree with comparable studies, which have shown that effective reinstatement of physical infrastructure is a key enabler of local community recovery (Ghanbarzadeh Ghomi et al. 2021). For the built environment to be effectively restored, there is a need to build-back-better in the post-disaster recovery reconstruction phase, by effectively restoring transportation systems and to make infrastructure more resilient (Wedawatta et al. 2018). The following narration reflects a lived experience from a male villager, from Nedziwa in Chimanimani:

Cyclone Idai was so severe that our road was badly affected from Nedziwa to Chimanimani. As a result of the damage to the road, the bridge at Nedziwa was also affected. The Nedziwa Bridge was heavily destroyed and it needed to be reconstructed since the river was now impassable (Male respondent, 44 years, Nedziwa, Chimanimani).

This comment clearly demonstrates that there is a need to reconstruct Nedziwa road, the bridge, and other structures to be better than the pre-disaster infrastructures. The reconstruction of resilient infrastructure following disasters has the advantage of creating safer and more sustainable communities. Communities such as Nedziwa also shared the experience of losing household property in the form of shelter, furniture, personal documents, children’s school books and stationery, as well as damage to cars, scotch-carts, and farming equipment (Table 2), among other belongings. These losses imply that homelessness was created, children’s education was interrupted, and, above all, the goal of delivering quality education was impacted. Post-disaster recovery programs in Chipinge and Chimanimani Districts should consider disaster risk reduction efforts that reduce disaster impact on households, and also support the drive towards quality education.

The impact of the cyclone on the environment and the loss of important community livelihoods mean that economic losses also were sustained in the two districts. Building-back-better would ensure that environmental and livelihood sustainability was attained, which would benefit future generations (Ghanbarzadeh Ghomi et al. 2021). The destruction inflicted on timber plantations and forests and the cyclone’s negative effect on wildlife species was extensive; losses to forests and wildlife are an economic loss that should be avoided in future. The impact on community livelihood resources water sources (boreholes, dams, wells, and springs), sugarcane and banana plantations, and livestock (Table 2), among others, suggests that water supply and food security were impacted. One female respondent from Ngaone Ward in Chipinge District said:

The cyclone left me in a poor state because my cattle herd, consisting of 6 beasts, were all destroyed through heavy flooding. Also, my banana plantation which was a source of food and income was affected. Right now, I no longer have any stable livelihood due to the devastation caused by the cyclone (Female villager, 56 years, Ngaone Ward, Chipinge).

This statement implies that community efforts to create sustainable livelihoods were also disturbed. Rural communities depended on their livestock, sugarcane and banana plantations, and other agricultural activities for survival. Our results resonate with previous studies, which found that building the resilience of rural communities in Chipinge and Chimanimani Districts is a productive way to achieve sustainable communities (Wright 2016). If build-back-better is not adopted, these communities are likely to suffer the same impact when confronted with future hazards. The section that follows evaluates the build-back-better considerations in the Cyclone Idai post-disaster recovery.

### Evaluating the Build-Back-Better Considerations in Chipinge and Chimanimani

The authors advocate a post-disaster recovery processes for Chipinge and Chimanimani that avoids a repeat of Cyclone Idai-scale losses in the future. Thus, building-back-better is more about reducing disaster risk and avoiding future vulnerability in these communities. This section evaluates the build-back-better considerations for the Cyclone Idai post-disaster recovery.

The DRR practitioners in FGDs highlighted the need for post-disaster recovery programs carried out by the government of Zimbabwe through existing Civil Protection Committees in partnership with other organizations. For this study, the recovery program is categorized under the four clusters: (1) reconstruction; (2) disaster risk reduction training; (3) livelihood revival; and (4) psychosocial support. Table 3 illustrates the four build-back-better clusters and their areas of focus. The programs involved are discussed separately in the following subsections.

**Table 3:** Cyclone Idai post-disaster recovery build-back-better clusters

Cluster category	Example of post-disaster recovery activity
Reconstruction cluster	(1) Reconstruction of the Nedziwa Bridge (2) Reconstruction and resurfacing of the Nedziwa–Chimanimani road (3) Reconstruction of the Changadzi–Chipinge road (4) Rebuilding of houses (5) Reconstruction of classroom blocks (5) Provision of tents as shelters
Disaster risk reduction training cluster	(1) Training of District Civil Protection Committees in community-based disaster risk reduction (2) Training of teachers and pupils in disaster risk reduction emergency drills
Livelihoods revival cluster	(1) Drilling of boreholes (2) Resuscitation of wells and water springs (3) Assistance to households with farming inputs
Psychosocial support cluster	(1)Establishment of trauma and counseling centers (Paidamwoyo clinic)

*Source* Authors’ construction from field data, 2020−2021

#### Reconstruction Cluster

The government and its partners embarked on an effort to rebuild damaged infrastructure. The FGDs conducted with DRR practitioners revealed that the infrastructure that was reconstructed included the school classroom blocks in both districts; the reconstruction of the 80 km stretch of Nedziwa–Chimanimani road; and, the rebuilding of the Nedziwa Bridge. The respondents indicated that the government was the main partner in the reconstruction process involving the road network. Some organizations, such as Africa Ahead, World Vision, and United Nations High Commissioner for Refugees (UNHCR) spearheaded the rebuilding of houses in the two districts. According to the practitioners, the UNHCR assisted the disaster survivors who were internally displaced. Unfortunately, many of the shelters (tents) that were provided by the UNHCR for temporary use were turned into permanent accommodations. This reconstruction in Chimanimani lacks a build-back-better character and does not support the expectations of Priority 4 of the Sendai Framework. Disaster survivors also revealed through interviews that they had turned the tents into permanent accommodations because post-disaster recovery processes failed to provide permanent shelter two years after the disaster. These results concur with disaster experience elsewhere (Dube et al. 2018; Wedawatta et al. 2018). Post-tsunami reconstruction in Nias, Indonesia lacked a comprehensive reconstruction plan; hence efforts to achieve the build-back-better were impeded (Haris et al. 2019). Post-disaster recovery in the form of tents cannot prevent future vulnerability to disasters in Chimanimani District.

According to the tenants, they have been using the tents since 2019 following the Cyclone Idai disaster. When this study was completed in March 2021, the occupants had used the tents as shelter for more than two years, suggesting that the reconstruction activities for the Ngangu community would hardly go beyond this stage. A previous study conducted in Tsholotsho District in Zimbabwe also revealed that people continued to live in tents several years after disaster impact (Dube et al. 2018). Should another cyclone visit the Ngangu community, they are likely to suffer the same disaster impact experienced during Cyclone Idai. Such temporary shelter is contrary to Sendai Framework’s focus on “build-back-better” recovery (Busayo et al. 2020). This suggests the need for effective strategies to construct permanent housing that incorporates the build-back-better ethos and moves on from emergency shelter/temporary housing. A previous study indicated that technologies such as offsite manufacturing could offer significant opportunities to build-back-better in contexts like these in a systematic and managed way (Thurairajah et al. 2019). In contrast to this failure in the Ngangu community, we observed that the rebuilt Nedziwa Bridge appeared to be stronger and better than the one destroyed by the cyclone, and thus Sendai compatible. These results agree with available data, which show that increased capabilities are required for managing the impact of disasters on the built environment (Adeniyi et al. 2017).

#### Disaster Risk Reduction Training Cluster

Disaster risk reduction training has been conducted in Chipinge and Chimanimani Districts as part of post-disaster recovery initiatives. We categorize this activity as the Disaster Risk Reduction Training Cluster (Table 3). Based on feedback from the FGDs, the DRR training was meant to build-back-better by equipping stakeholders with DRR skills, understanding of disasters, and emergency knowledge. Available data have also shown that understanding disasters means acknowledging that disasters emanate from local and socially produced vulnerabilities and failures as natural hazards (Perry and Quarantelli 2005; Oliver-Smith et al. 2017). The stakeholders to undergo DRR training were drawn from the District Civil Protection Committees (CPCs), which consist of employees from government departments, humanitarian agencies, and NGOs. The DRR practitioners indicated that they have embarked on the training of the stakeholders in Chipinge and Chimanimani Districts as a capacity building effort. The CPCs were to be upgraded through the disaster risk reduction curriculum, with a specific focus on community-based disaster risk reduction. The training of DRR practitioners is a build-back-better initiative in the spirit of the Sendai Framework. Disaster risk reduction practitioners identified World Vision as the lead entity in the training of the CPC members in the districts. One practitioner, representing World Vision, narrated how the training was progressing:

Our training program centered on community-based disaster risk reduction is targeting members of the District CPCs so that we capacitate them in managing hazards at the community level. World Vision is leading in this project and we wish to see all members of the District CPCs getting basic DRR training. We, therefore, expect the CPC members to go back and train community members on what they would have learnt from this exercise (Male, practitioner, 40 years, World Vision, Chimanimani).

Apart from providing DRR training to members of the CPCs, training was also done in schools. According to the FGD respondents, this DRR training also targeted teachers and pupils in primary and secondary schools. The DRR curriculum was based on emergency drills and was carried out under the program named Education in Emergencies—Education Access, Disaster Preparedness and Child Protection. This training program for schools was driven by a consortium of organizations including World Vision, Plan International, and Save the Children, which was ably supported by the European Union. The main focus of the program was to inculcate DRR knowledge, skills, and procedures to both the teachers and pupils so that they would be more resilient against future flooding events or related hazards. These results resonate with the view of Weichselgartner and Kelman (2015), who urged that a combination of ideas among stakeholders is needed to produce an encompassing strategy for building disaster resilience. Pre-Ida DRR training had already been done in many primary schools that included the Mbire, Rusitu Valley, Tanganda, Chisuma, and Rimaye primary schools. Secondary schools that had received training included Mapungwana, Nyaututu, and Tuzuka. The capacity building of the CPC members, community members, teachers, and children is a step in the right direction that supports build-back-better and Sendai Framework endeavors, as the program prepared the stakeholders for future hazards.

#### Livelihoods Revival Cluster

The Livelihoods Revival Cluster (Table 3) focused on the resuscitation of livelihoods. The recovery program entailed drilling new boreholes, repairing malfunctioning old boreholes, and resuscitating natural springs, all activities in line with the build-back-better concept and Sendai Framework Priority 4. Organizations such as GOAL and UNICEF (United Nations International Children’s Emergency Fund) were leading in the provision of clean water through the resuscitation of water sources. Instead of just restoring the malfunctioning boreholes, new boreholes were drilled. This ensured that there was an improvement in water supply, hence building-back-better was realized through new boreholes. Continued access to safe and clean drinking water is an aspect of effective recovery that supports the Sendai Framework and building-back-better following a disaster event. The disaster-impacted communities also were assisted with crop seed and farming equipment with which to revive their farming livelihoods. Information from the practitioners revealed that not every disaster-impacted household had access to farming equipment assistance. Only a few, who were perceived to be needier, were assisted, although humanitarian assistance should be guided by the need of the disaster-impacted households, effective post-disaster recovery should also focus on assisting a majority of those impacted. Hence, building-back-better and the Sendai Framework focus were compromised as more survivors still needed to be assisted.

#### Psychosocial Support Cluster

The Psychosocial Support Cluster (Table 3) was an attempt to render psychological, social, and emotional support to Cyclone Idai-impacted communities. Counseling centers were established at selected clinics in the two districts so that counseling and emotional support could be rendered to those who suffered cyclone impact. Some people were traumatized by seeing dead bodies and losing their loved ones to the cyclone. Others had undergone frightening experiences in surviving the Cyclone Idai-induced flooding. For these communities to effectively recover from the impact and to build-back-better, community centers were needed in the districts where counseling was to be provided. One major psychosocial support center was created at Paidamwoyo clinic in Chipinge. But there were too few in the two districts to meet expectations of the build-back-better concept and align properly with Priority 4 of the Sendai Framework.

Overall, build-back-better considerations for the post-disaster recovery clusters in Chipinge and Chimanimani Districts seem to be lacking. This is a common observation in developing countries (Jogia and Wedawatta 2019) as limited resources available are often allocated towards reconstruction. The subsection that follows focuses on the build-back-better lessons learnt from the Cyclone Idai disaster.

### Build-Back-Better Lessons Learnt from the Cyclone Idai Disaster in Chipinge and Chimanimani

From the perspectives of disaster risk reduction practitioners and communities in Chipinge and Chimanimani Districts, it is clear that there are important early and ongoing lessons to be learnt from the Cyclone Idai disaster event. From the FGDs carried out with the practitioners and the interviews done with the community members, four major build-back-better lessons emerged for this study: (1) lack of understanding of disaster vulnerability and risk can lead to huge losses; (2) building-back-better should not be an option, but a mandatory post-disaster recovery expectation; (3) building-back-better creates safer, resilient, and more sustainable communities; and (4) investment in DRR training should be a priority.

#### The Need to Understand Disaster Vulnerability and Risk

Focus group discussion participants agreed that the huge losses caused by Cyclone Idai-induced floods revealed gaps in understanding community disaster vulnerability and associated risks. The practitioners’ views were that the CPCs and the communities had a limited perception of the vulnerable conditions exposed by the flood hazards, despite the location of some communities near rivers and in low-lying areas. Sun and Faas (2018) insist that to understand disasters, communities must not only think about the hazards that might affect them, but also about the different levels of their vulnerability. There existed a low-risk perception in many communities, which prevented anticipation that any potential disaster losses were associated with settlement in hazardous areas. One member of the CPC stated that although an early warning was given to communities through various forms of media, most people did not take the information seriously. One DRR practitioner said:

When the cyclone was imminent, an early warning was provided to the communities through newspapers, televisions and WhatsApp group platforms. However, only a few people managed to relocate to safer areas, whilst those who were skeptical of the warning remained behind. Those who decided to stay behind are the ones who suffered the worst impact of Cyclone Idai because they were caught unprepared. Unfortunately, many people who were affected lost their lives (Male, CPC member, 48 years, Chipinge).

At least some people within the communities had information about the hazard, their vulnerability, and risk, but the information was not taken seriously. Some of the information was conveyed through print, television sets, and WhatsApp platforms. These media platforms are not an ideal strategy to convey messages to the communities in rural areas, since most of the people in rural areas have limited access to such media platforms. Vulnerability in Chipinge and Chimanimani Districts is determined by social systems, and not by natural hazards (Tierney 2007). Public education and awareness campaigns by DRR practitioners could have been more ideal, since these techniques provide face to face interactions with community members. One of the lessons from Cyclone Idai is the need to understand disaster vulnerability and risk.

#### Build-Back-Better as a Mandatory Post-Disaster Recovery Exercise

Both the practitioners and community members in our study expressed the feeling that there was a compelling need to build-back-better. They regarded enhanced post-disaster recovery as a mandatory, non-optional exercise that ensures future risks are avoided or reduced and resilience is increased. Previous studies indicate that if only restored to pre-disaster standards, communities would suffer the same difficulties if exposed to another disaster event (Mannakkara et al. 2018). If build-back-better is taken as a mandatory standard, communities in Chipinge and Chimanimani would benefit in that they would better able to limit disaster losses in the future. Should disaster losses be reduced, the same communities might also see the fulfillment of their development goals. Above all, restored livelihoods would make possible good health and better well-being.

#### Building-Back-Better Creates Safer, Resilient, and Sustainable Communities

Respondents also foresaw safer, resilient, and more sustainable communities if post-disaster recovery cluster goals were effectively implemented. Effective reconstruction entails the rebuilding of strong or improved infrastructure such as damaged shelters, roads, bridges, and school classrooms post-disaster. This result agrees with a previous study in Asia, which found that the post-earthquake recovery through housing reconstruction was seen as an opportunity to create safer and more sustainable communities that minimize casualties in future (Cutter 2015). Study participants also regarded safe, resilient, and sustainable communities as a logical outcome of relevant DRR training for community members and practitioners and the effective revival of community livelihoods. The resuscitation of water sources, adoption of new measures for farming, and protection of sugarcane and banana plantations were advocated as crucial development initiatives. By rendering psychosocial support to the disaster survivors, effective post-disaster recovery is augmented, since such support diffuses trauma and mitigates thinking of bad experiences.

#### Taking Investment in Disaster Risk Reduction Training as a Priority

Finally, prioritizing investment in DRR training was seen as one of the lessons to be learnt from the Cyclone Idai disaster experiences. While the training of CPCs, community members, teachers, and school children was seen as a noble idea, some respondents felt that such training was not done regularly enough. According to study respondents, training programs to increase stakeholder capabilities in disaster risk reduction issues should be well supported with funds and other resources; funding constraints do not motivate the stakeholders and communities to take disaster prevention measures. Previous research also showed that lack of financial resources significantly hinders the ability to better prepare for future hazards, for instance, by moving out of vulnerable areas and making homes safer (Wedawatta et al. 2016). The respondents’ opinions were that such training programs should be carried out regularly. Sawada and Takasaki (2017) found that DRR activities must be supported with funds, which should be distributed to disaster-affected people and locations based on the level of local vulnerability and disaster impact. Our frequency-critical respondents indicated that DRR training content was adequate and contributed significantly to their understanding of local hazards, vulnerability, and risks.

We believe that the provision of regular training on DRR ensures that long-lasting knowledge is inculcated into the stakeholders. During the recovery process after the 2005 earthquake in Pakistan, Haris et al. (2019) noted that build-back-better suffered from a lack of skilled labor. Hence, regular training has the potential to address future vulnerabilities and avoid disaster losses. Very important lessons were learnt from the Cyclone Idai disaster, and, if such lessons are incorporated into local training, future disaster losses can be reduced.

## Conclusions

Our study concludes that cyclones can cause a severe impact on the communities living with vulnerability to hazards and risks. Such communities continue to interact with hazards by living in disaster-prone areas, thereby worsening their state of vulnerability. Disasters impact most communities with limited vulnerability and risk perception, and little disaster knowledge. We also have determined that build-back-better considerations for Cyclone Idai recovery were inadequate and that more needs to be done in this respect. We propose that build-back-better should be regarded as a mandatory post-disaster recovery principle, and not as an optional feature of the recovery process. Moreover, important lessons can be drawn from the Cyclone Idai disaster event. If the opportunities presented by building-back-better are to be fully exploited, clear strategies for and a focused approach to emergency response and recovery is needed. While some crucial opportunities have been missed during short to medium term recovery in Chipinge and Chimanimani, there still are opportunities to build-back-better in medium to long term recovery of affected communities.

We recommend the government, its partners, and community members adopt the build-back-better concept in their post-disaster recovery programs. The importance of community cohesion and social capital in disaster risk reduction cannot be ignored. Humanitarian assistance to the disaster-impacted communities should have a specific focus on all those who have been impacted. Continuous training programs for practitioners and communities are required for sustained disaster knowledge and awareness. Future research should consider the role of indigenous knowledge systems in better rebuilding disaster-impacted communities. Further research can be undertaken to understand how the initial decisions on building-back-better influence long-term recovery and reestablishment of affected communities.
